# *Lactococcus lactis* Endocarditis in an Immunocompromised Patient

**DOI:** 10.1155/2022/3845679

**Published:** 2022-11-08

**Authors:** Austin Mitchell, Sarah Gorn, Alex Cheung, Spogmai Khan, Cynthia Anneski

**Affiliations:** Mountain Vista Medical Center, Midwestern University, 10238 E Hampton Ave Ste 200, Mesa, AZ 85209, USA

## Abstract

*Lactococcus lactis* infections are rarely reported in the medical literature. *L. lactis* is a commonly used fermenting agent which may be difficult to identify with common microbiology identification processes. This factor may contribute to its lack of recognition in medical journals. We report a case of an immunosuppressed 80-year-old female with *L. lactis* bacteremia, subsequently, found to have aortic valve vegetation, who responded clinically to a six-week duration of ceftriaxone therapy. Afterward, a brief updated literature review is presented on *L. lactis* infections.

## 1. Introduction

*Lactococcus lactis*, formerly known as *Streptococcus lactis*, is a Gram-positive coccus and a facultative anaerobe [1]. *L. lactis* is commonly used in the dairy industry as a fermenting agent; however, there are some case reports of *L. lactis* causing human infections [2]. We hereby report a case of an immunocompromised patient with infective endocarditis secondary to *L. lactis* infection with known consumption of processed cheese. Following the case report, we present a brief updated literature review with known cases since 2018 with their treatments and outcomes included.

## 2. Case Presentation

An 80-year-old Caucasian female with a past medical history of rheumatoid arthritis (on current immune suppressive therapy with etanercept and hydroxychloroquine), atrial fibrillation, type 2 diabetes mellitus, hypertension, hyperlipidemia, chronic lymphedema, and prior breast cancer (currently in remission) presented with generalized weakness. She endorsed associated increased bilateral lower extremity swelling, shortness of breath, dry cough, and orthopnea. She denied any recent subjective fevers. Vitals were significant for a heart rate of 98. All other vital signs were nonsignificant including temperature. Her physical exam was significant for coarse breath sounds bilateral, trace bilateral pitting edema, and a pustular area with erythema on the second digit of her right foot. Specifically, on cardiac examination, no murmur was appreciated; however, a regular rate with irregular rhythm was noted. Chest radiography showed bilateral pleural effusions and bilateral lower lobe consolidation. Right foot radiography showed concern for osteomyelitis. A right foot magnetic resonance image was positive for cellulitis, however, negative for osteomyelitis. Transthoracic echocardiogram showed an ejection fraction of 50–55% with mild mitral valve regurgitation and stenosis and severe tricuspid regurgitation. Blood cultures were collected in the Emergency Department upon arrival. During the course of her hospitalization, she was aggressively diuresed and started on intravenous ceftriaxone and azithromycin for possible community-acquired pneumonia. On the first day of her admission, her symptoms improved; however, one out of two blood cultures resulted positive for *Streptococcus* species. On day two, both cultures were positive. Intravenous azithromycin was discontinued while intravenous ceftriaxone was continued with the addition of intravenous vancomycin. On day four, blood cultures had the final growth of *Lactococcus lactis*. This was identified by Verigene nucleic acid testing. Unfortunately, the Microbiology Department was unable to perform susceptibility on *L. lactis*. Further investigation as to the source of infection was initiated. The patient's family was asked to bring in her probiotics which were inspected and did not include *L. lactis* species. CT scans of the chest, abdomen, and pelvis with contrast were obtained which were positive for possible proctitis. A colonoscopy revealed multiple tubular adenomas and was otherwise negative. A transesophageal echocardiogram noted a trileaflet calcified aortic valve with small mobile vegetation attached to the coronary cusp. In a further interview, the patient confirmed she was lactose intolerant, however, admitted to occasional string cheese consumption and denied other milk products, including unpasteurized milk products. Antibiotics were deescalated to ceftriaxone alone. Repeat blood cultures were obtained which showed no growth after five days of antimicrobial therapy. Ultimately, a peripherally inserted central catheter line was placed and the patient was discharged on intravenous ceftriaxone for six weeks. She received a total of eleven days of treatment with ceftriaxone inpatient and four days of vancomycin treatment inpatient until antibiotics were de-escalated.

The patient was readmitted six days later for an unrelated issue wherein blood cultures were obtained and remained negative.

## 3. Discussion

A review of current literature shows there have been a total of 41, including this case, reported infections with *L. lactis*. A thorough review was provided in 2018 and since then, there have been four new cases of *L. lactis* infection, including our own [2–40]. Endocarditis continues to be the most prevalent, with nine reported cases [3–10]. Our report is only the second reported case of a female with *L. lactis* endocarditis and the first to be reported with an underlying rheumatologic disorder on immune-suppression therapy. While *L. lactis* was not listed in the ingredients of her probiotic, it is possible this may have been a small component of the ingredients that were not listed.

Since 2018, the reported cases of *L. lactis* include the following three reports. One infection occurred in a patient with known exposure from their probiotic supplement and subsequently developed bacteremia [38]. This patient was treated with ertapenem and amoxicillin and instructed to discontinue the probiotics. A follow-up showed improvement in clinical status. Another case occurred as chorioamnionitis in a patient with known exposure to unpasteurized buttermilk [39]. She was treated with amoxicillin 1 gram, three times daily for 10 days. Lastly, an 18-year-old male was diagnosed with a brain abscess which cultures resulted in *L. lactis* [40]. This patient was treated with ceftriaxone and was symptom-free on a six-monthfollow-up.

## 4. Conclusion

Endocarditis is a fairly rare disorder, affecting 3–10 people per 100,000 per year. The most common bacteria responsible are streptococci and staphylococci. For native valve infective endocarditis, the standard length of treatment requires four to six weeks of antibiotics after the first day of negative blood cultures. Our case highlights a rare pathogen causing bacteremia, *L. lactis*. The majority of *L. lactis* bacteremic infections arise from infective endocarditis, we recommend pursuing a transesophageal echocardiogram for workup when this pathogen results in blood culture.

## Data Availability

The health record data used to support the findings of this case report are restricted in order to protect patient privacy. Appropriate certain health record data are included verbatim within the article. This case report also provides a discussion of *Lactococcus lactis*. The data used in the discussion were found in peer-reviewed journals and previously published case reports. Appropriate citations and references are included within the article.
